# Combenefit: an interactive platform for the analysis and visualization of drug combinations

**DOI:** 10.1093/bioinformatics/btw230

**Published:** 2016-04-25

**Authors:** Giovanni Y. Di Veroli, Chiara Fornari, Dennis Wang, Séverine Mollard, Jo L. Bramhall, Frances M. Richards, Duncan I. Jodrell

**Affiliations:** ^1^CRUK Cambridge Institute, University of Cambridge, Cambridge, UK; ^2^Early Clinical Development, Innovative Medicines and Early Development Biotech Unit, AstraZeneca, Cambridge, UK; ^3^Bioinformatics, Oncology Innovative Medicines, AstraZeneca, Cambridge, UK

## Abstract

**Motivation:** Many drug combinations are routinely assessed to identify synergistic interactions in the attempt to develop novel treatment strategies. Appropriate software is required to analyze the results of these studies.

**Results:** We present Combenefit, new free software tool that enables the visualization, analysis and quantification of drug combination effects in terms of synergy and/or antagonism. Data from combinations assays can be processed using classical Synergy models (Loewe, Bliss, HSA), as single experiments or in batch for High Throughput Screens. This user-friendly tool provides laboratory scientists with an easy and systematic way to analyze their data. The companion package provides bioinformaticians with critical implementations of routines enabling the processing of combination data.

**Availability and Implementation:** Combenefit is provided as a Matlab package but also as standalone software for Windows (http://sourceforge.net/projects/combenefit/).

**Contact:** Giovanni.DiVeroli@cruk.cam.ac.uk

**Supplementary information:** Supplementary data are available at *Bioinformatics* online.

## 1 Introduction

The combination of drugs is an important strategy to treat various diseases. The goal is to increase efficacy and, through the avoidance of overlapping toxicity, improve the therapeutic index of treatment (Long *et al.*, 2014). At the discovery stage, research often focuses on the identification of synergistic target effects using *in vitro* systems (Lehár *et al.*, 2009; Roemer and Boone, 2013; Sun *et al.*, 2013). Large scale combination screens testing multiple pairwise combinations of drugs across different concentration ranges and cell lines are being performed with increasing frequency (He *et al.*, 2015; Held *et al.*, 2013; Mathews Griner *et al.*, 2014). Typically, the dose–response data generated during these experiments are then analyzed in terms of synergistic or antagonistic effects. Free software were developed in the past but these are limited in terms of data handling and available options, particularly given high throughput screens (HTS) requirements (http://www.combosyn.com/; Dressler *et al.*, 1999; Prichard and Shipman, 1990). Two recent commercial software packages offer advanced features such as automated analyses, choice from a variety of models, quality curve-fitting, variety of graphical displays and metrics quantification (Chalice http://cwr.horizondiscovery.com; Genedata https://www.genedata.com). There is a lack of free, advanced, scalable, software for the analysis of drug combinations. As part of the growing effort in the search of effective combinations, we present here Combenefit (‘*Combinations Benefit*’), a new software tool that enables the visualization, analysis and quantification of drug combination effects, in terms of synergy and/or antagonism, for single combination experiments but also HTS as per commercial packages (Table 1).

**Table 1.** Features of available software for drugs combinations analyses. L: Loewe, B: Bliss, H: HSA, DR: Dose–response curve, M: Matrix view, C: contours, S: surfaces, I: Isobolograms, SM: synergy mapped to dose–response surfaces, F: Fa related curves, see (Chou, 2010). Note: we have not included CombiTool (Dressler *et al.*, 1999) because it does not appear to be available anymore

	Combenefit	Chalice^TM^	Genendata Screener^®^	CompuSyn	MacSynergy™ II
Available	Free	Commercial	Commercial	Free	Free
Source code	Yes	No	No	No	Spreadsheet
Replicates	Yes	Yes	Yes	No	Yes
Fitting (Hill model)	3 params	3 params	3 params + baseline	2 params (no max)	No
Synergy models	L,B,H (+planned)	L,B,H and 2 others	L,B,H	L based	B
Scores/metrics	Several	Several	Several	No	No
Graphics	DR,M,C,S,SM	DR,M,C,I	DR,M,C,I	DR,I,F	C,S
Data input	File	File	File	Manual	Manual
HTS suitable	Yes	Yes	Yes	No	No

## 2 General overview

Combenefit implements a surface approach, where *in vitro* experimental data is compared to mathematical models of dose–responses for non-synergistic combinations (also termed additive or independent combinations; Greco and Bravo, 1995). The three classical models, namely the Loewe (Loewe and Muchnick, 1926; Loewe, 1953), the Bliss (Bliss, 1939; Webb, 1963) and the Highest Single Agent (HSA; Mathews Griner *et al.*, 2014; Tan *et al.*, 2012) models have been incorporated. These models have been used extensively in the literature, with different fields having their preference and sometimes alternative names for these same models (Greco and Bravo, 1995; Odds, 2003). It is important to use these models in a consistent way and to provide results in the context of the model used. Additionally, our own group is currently developing a new general model which, together with any other future feature, will be incorporated into Combenefit.

**Fig. 1.**
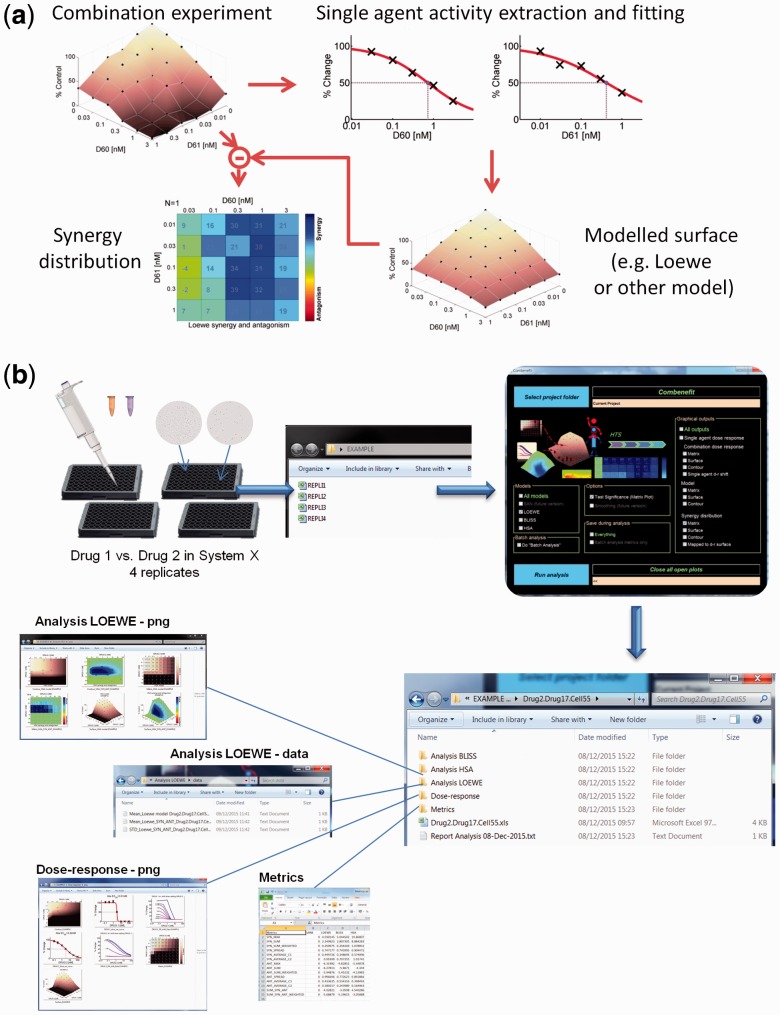
Analysis and visualization of drug combinations with Combenefit. (**a**) Illustration of analysis principle. (**b**) Illustration of automated processing (Color version of this figure is available at *Bioinformatics* online.)

The approach implemented in Combenefit can be summarized as follows (Fig. 1a). The experimental dose–response surface that delineates combination effects in concentration space, is first read by the software as a matrix of % of the control value across concentrations. Single agent effects are extracted from this data and fitted with a dose response curve. Based on the two single agent dose response curves, a model-based combination dose–response surface is derived. This surface provides a ‘reference’ dose–response surface for a non-synergistic (additive/independent) combination, whose characteristics are determined by the selected model (Loewe, Bliss, etc.). The experimental combination dose response surface is then compared to the model-generated one, resulting in a synergy distribution in concentration space. This synergy distribution can be further summarized via various metrics as described below.

## 3 Using Combenefit

The software is available as a standalone application for Windows or as a Matlab package. The standalone application can be installed automatically and is then directly accessed through its own icon. The Matlab package needs to be saved in a dedicated folder which is then accessed via Matlab by calling the *Combenefit* routine contained in the package. Laboratory scientists can quickly and easily analyze their data using either version. Bioinformaticians can use the code provided in the Matlab package to facilitate their own projects involving drugs combinations.

### 3.1 Typical analysis

A typical analysis is performed as follows. Upon completion of experiments, the results of a specific combination (including replicates if available) are saved in .xls files (the data is ordered with the first drug in columns and the second in rows; a template is provided). Once Combenefit is launched, the project folder containing all experimental .xls files is selected (Fig. 1b). Then, one or more reference models can be selected. Other options and graphical outputs can be chosen as per requirements/preferences. The graphical outputs consist of: (i) the single agent dose response data and its fitting, (ii) the combination dose response (Four different displays), (iii) the model-generated reference combination dose response, i.e. the prediction of effect if the drugs are not synergistic (Three different displays) and (iv) the resulting synergy distribution (Three different displays). Additionally, a fourth graphic mapping the synergy distribution onto the dose–response surface can also be displayed to facilitate interpretation. All figures, as well as corresponding data, fitting parameters and synergy metrics (see below) can be saved in the project folder. Figures are saved as high quality .png or .pdf files which can be directly used in publications. A user’s guide with step-by-step guidance for installing and running the software is available online.

### 3.2 Metrics and batch analysis

The analyses described above result in a synergy distribution for the combination being processed. To facilitate comparison across experiments, or for HTS applications, it is useful to summarize the resulting synergy and/or antagonism distribution using one or more metrics. Combenefit provides a set of metrics (or scores) which captures information about the synergy distribution. These include metrics such as the maximum synergy, the integrated and the weighted integrated synergy and concentration value at which synergy is most dense (for a full list of metrics and their formulation, please see the Supplementary Material).

During HTS, large volumes of combinations and cell lines are investigated using automated technology. Combenefit can be used to analyze the large amount of data derived with these screens. Using Combenefit, the user selects the folder containing all experiments (each in an individual sub-folder) and then selects the ‘Batch Analysis’ option. Upon running the analysis, Combenefit will batch process in a sequential way all the folders. The results of the analysis are summarized in a table containing all the experiments in rows and the metrics in columns. Typically, the highest hits are then visually inspected using Combenefit’s graphical outputs to improve understanding of the data and prioritize drug combinations. Combenefit has been recently used to generate the AstraZeneca dataset (∼11 500 combinations) provided for the current AstraZeneca-Sanger Drug Combination Prediction DREAM challenge (http://dreamchallenges.org/).

In summary, we have described the overall implementation and functioning of Combenefit, new software which offers advanced graphical capabilities and allows for model-based quantification of drug combinations in single and high-throughput settings. We hope that this new tool will continue to improve and will help many scientists in a variety of fields involving combination data analyses.

## Supplementary Material

Supplementary Data
