# Supplementary material for: Combenefit: an interactive platform for the analysis and visualization of drug combinations
Source: Bioinformatics. 2016 Apr 25;32(18):2866–8. doi: 10.1093/bioinformatics/btw230 (PMC5018366; doi:10.1093/bioinformatics/btw230)
Supplement: Supplementary Data [file supp_32_18_2866__index.html]

Combenefit: an interactive platform for the analysis and visualization of drug combinations — Combenefit: an interactive platform for the analysis and visualization of drug combinations — Supplementary Data 

# Combenefit: an interactive platform for the analysis and visualization of drug combinations

## Supplementary Data

files

- Supplementary Data - docx file
