# Supplementary material for: Combenefit: an interactive platform for the analysis and visualization of drug combinations
Source: Bioinformatics. 2016 Apr 25;32(18):2866–8. doi: 10.1093/bioinformatics/btw230 (PMC5018366; doi:10.1093/bioinformatics/btw230)
Supplement: Supplementary Data [file supp_btw230_supplementary_material.docx]

Supplementary material for:

Combenefit: An interactive platform for the analysis and visualisation of drug combinations

Giovanni Y. Di Veroli,^12^ Chiara Fornari,^1^ Dennis Wang,^3^ Séverine Mollard,^1^  Jo L. Bramhall,^1^  Frances M. Richards,^1^ Duncan I. Jodrell^1^

^1^ CRUK Cambridge Institute, University of Cambridge, UK

^2^ Early Clinical Development, Innovative Medicines and Early Development Biotech Unit, AstraZeneca-Cambridge

^3^ Bioinformatics, Oncology Innovative Medicines, AstraZeneca, UK

**Synergy distribution**

We consider the hypothetical combination of two agents A and B at concentrations *a* and *b* respectively. The effectiveness of a drug combination can be assessed in terms of the amount of "extra-effect" that is obtained when combining the drugs. Thus effects can be decomposed as following:

$$E\left( a,b \right)=R\left( a,b \right)+S(a,b)$$

Where *E(a,b)*is the observed effect, i.e. what we actually measure during the experiment. *R(a,b)* is the reference effect, often termed additive or independent (depending on the model used), i.e. a baseline which should be obtained in an experiment when the combination does not amplify or reduce cell kill. *S(a,b)* is the amount of extra-effect, also termed synergistic effect (when this extra-effect is negative, it is termed antagonistic). In order to identify synergy or antagonism, the combined reference effect *R(a,b)* is first derived based on single agent dose-response curves. Reference surfaces depend on the mathematical model which is used to define non-synergistic effects.

**Reference models**

The classical Loewe, Bliss and HSA models were implemented as follows.

*Loewe model*

For the Loewe model, the reference effect for the combination *(a,b)* is calculated by finding two doses *a_U_* and *b_U_* such that:

$$E_{A}\left( a_{u} \right)=E_{B}(b_{u})$$

And for which the isobole equation is verified:

$$\frac{a}{a_{U}}+\frac{b}{b_{U}}=1$$

These two equations are solved numerically for *(a_u_,b_u_).* The numerical solution is used to define the reference effect as:

$$R_{AB\_Loewe}\left( a,b \right)=E\left( a_{u} \right)=E_{B}(b_{u})$$

Because monotonic dose response curves are considered, if a solution exists, this solution is unique (i.e. the optimization process finds a global minimum). Note that, if for instance A has lower efficacy than B, then a solution does not exist for concentrations *b* high enough such that *E_B_(b)<min(E_A_)*. Thus, applicability of the Loewe model is in principle limited.

For cases where the original isobole’s equation of the Loewe model cannot be used due to differences in maximum effects, we developed an extension of the Loewe modelc as follows. Indeed, it can always be safely assumed that very high concentrations of A induce effects greater than observed efficacy (all substances are toxic at concentrations high enough). Thus, if we consider a concentration *a_u_* which is great enough, then the ratio *a/a_u_* becomes infinitely small and according to the isobole equation we obtain:

$$\frac{b}{b_{U}}=1$$

Thus, for concentrations *b* that induces effects beyond A’s observed maximum effect, we extend the Loewe model by defining the following reference:

$$R_{AB\_Loewe}\left( a,b \right)={E\left( b_{u} \right)=E}_{B}(b)$$

*Bliss model*

For the Bliss model, the reference effect for the combination *(a,b)* is obtained by taking the product of the effects at these concentrations:

$$R_{AB\_Bliss}\left( a,b \right)=E_{A}\left( a \right){\cdot E}_{B}(b)$$

In our case, the effect is defined as the fraction of unaffected control population (e.g. E(a=1nM)=0.8, means that 1nM of drug A reduces the expected population of cells by 20% - see also the online user’s guide on how to prepare the data).

*HSA model*

For the HSA model, the reference effect for the combination *(a,b)* is obtained by taking the greatest effect (lowest residual compared to control) between the two drugs as single agents:

$$R_{AB\_HSA}\left( a,b \right)={MIN(E}_{A}\left( a \right),E_{B}(b))$$

**Single agent dose response and statistical analysis**

The reference surface depends on the selected synergy model and is evaluated from the dose-response curves of each of the two agents. These dose-response curves are generated via fitting to a Hill equation (also called logistical sigmoidal function) with varying maximum effect:

$$HILL\left( X=x \right)=1+\frac{E_{c\infty}-1}{1+{({IC}_{50}/x)}^{H}}$$

 The parameters *IC_50_*, *H* and *E_c∞_* characterize the concentration where half of the effect is obtained, the slope of the dose-response curve, and the plateau effect (i.e. asymptote value) respectively. In practice, the inclusion of a varying plateau value in this formulation enables the appropriate fitting of most single agent dose-response curves. During the optimization process, either the trust-region-reflective (in which case a gradient function is provided) or bounded simplex algorithms are employed to minimize the following function (maximum likelihood approach):

$$F{(E}_{c\infty},{IC}_{50},H)=\sum_{i=1}^{n} \sum_{j=1}^{p} \left( \frac{E_{i}\left( c_{j} \right)-HILL(c_{j},E_{c\infty},{IC}_{50},H)}{\sigma_{j}} \right)^{2}$$

Where *c_j_* corresponds to the j^th^ concentration value for agent A or B (*p* concentrations), *E_i_(c_j_)* corresponds to the measured effect for concentration level *c_j_* and replicate *i* (*n* replicates), and *σ_j_* is the standard deviation obtained for all the measures at concentration *c_j_*. This fitting approach, which accounts for statistical variation, allows checking for goodness of fit (when *n > 1*). If goodness of fit is too low, a warning message is displayed by the software. The interpolated *Absolute half maximal effective concentration (Abs EC_50_*) is provided on the displayed dose-response curves. *Abs EC_50_* is defined as the concentration (or dose) which reduces the control (100% Control - no drug) by 50%. Note that this is different from the *IC_50_*, which corresponds to 50% of the maximum effect *E_c∞_*. The *IC_50_* exists independently of the actual value of the maximum effect, while the Abs *EC_50_* does not exist if *E_c∞_* is less than 50%. The interpolated *Relative 95% effective concentration (Rel EC_95_*) is given if efficacy is lower than 50%. *Rel EC_95_* is defined as the concentration (or dose) which gives 95% of the agent’s maximum effect.

The resulting dose-response curves are used to generate the dose-response surface for the reference model. The software compares the experimental surface to the modelled one and attributes a percentage score. In the matrix display, if more than one replicate is provided, the software colours each synergy level only if the result is significant following a one‑sample t‑test (* p-value <5x10^-2^_;_ ** p-value <10^-3^, *** p-value <10^-4^), with the number of replicates shown on the left top corner of the matrix display. Note that this test should be used with caution when only a low number of replicates are available.

**Synergy metrics**

Several metrics which summarize features of the synergy distribution are provided, as listed in the following table:

| *Metric name* | *Metric formula* |
| --- | --- |
| ***Explanation of metric*** | |
| SYN_MAX | $\boldsymbol{max(S(a,b))}$ |
| **This is the maximum level of synergy observed.** | |
| ANT_MAX | $\boldsymbol{min(S(a,b))}$ |
| **This is the maximum level of antagonism observed.** | |
| SUM_SYN_ANT | $\iint_{\boldsymbol{min}\left( \boldsymbol{a}>0 \right)\boldsymbol{,min(b}>0)}^{\boldsymbol{max}\left( \boldsymbol{a} \right)\boldsymbol{,max(b)}} \boldsymbol{S}\left( \boldsymbol{a,b} \right)\boldsymbol{\cdot dlog(a)\cdot dlog(b)}$ |
| **This is the sum of synergy and antagonism observed in concentration logarithmic space. For instance, an integrated synergy of 50 is equivalent to an extra synergistic effect of 50% which is spread over a square of 1 log x 1 log in the 2-d log-concentration space. Both antagonistic and synergistic effects are considered in this metric.** | |
| SUM_SYN_ANT_WEIGHTED | $\iint_{\boldsymbol{min}\left( \boldsymbol{a}>0 \right)\boldsymbol{,min(b}>0)}^{\boldsymbol{max}\left( \boldsymbol{a} \right)\boldsymbol{,max(b)}} \boldsymbol{(1-E(a,b)/100)\cdot S}\left( \boldsymbol{a,b} \right)\boldsymbol{\cdot dlog(a)\cdot dlog(b)}$ |
| **The integrated weighted synergy incorporates a weight based on the dose response which bias the total score towards synergy achieving highest effect. Hence, a synergy of 50% leading to a combined full effect (100%) will have more weight than if the corresponding effect was only 20 or 30%. Both antagonistic and synergistic effects are considered in this metric.** | |
| SYN_SUM | $\iint_{\boldsymbol{min}\left( \boldsymbol{a}>0 \right)\boldsymbol{,min(b}>0)}^{\boldsymbol{max}\left( \boldsymbol{a} \right)\boldsymbol{,max(b)}} \boldsymbol{max(S}\left( \boldsymbol{a,b} \right)\boldsymbol{,0)\cdot dlog(a)\cdot dlog(b)}$ |
| **Same as SUM_SYN_ANT, but antagonistic effects are not considered.** | |
| SYN_SUM_WEIGHTED | $\iint_{\boldsymbol{min}\left( \boldsymbol{a}>0 \right)\boldsymbol{,min(b}>0)}^{\boldsymbol{max}\left( \boldsymbol{a} \right)\boldsymbol{,max(b)}} \boldsymbol{(1-E(a,b)/100)\cdot max(S}\left( \boldsymbol{a,b} \right)\boldsymbol{,0)\cdot dlog(a)\cdot dlog(b)}$ |
| **Same as SUM_SYN_ANT_WEIGHTED, but antagonistic effects are not considered.** | |
| ANT_SUM | $\iint_{\boldsymbol{min}\left( \boldsymbol{a}>0 \right)\boldsymbol{,min(b}>0)}^{\boldsymbol{max}\left( \boldsymbol{a} \right)\boldsymbol{,max(b)}} \boldsymbol{min(S}\left( \boldsymbol{a,b} \right)\boldsymbol{,0)\cdot dlog(a)\cdot dlog(b)}$ |
| **Same as SUM_SYN_ANT, but synergistic effects are not considered.** | |
| ANT_SUM_WEIGHTED | $\iint_{\boldsymbol{min}\left( \boldsymbol{a}>0 \right)\boldsymbol{,min(b}>0)}^{\boldsymbol{max}\left( \boldsymbol{a} \right)\boldsymbol{,max(b)}} \boldsymbol{(1-E(a,b)/100)\cdot min(S}\left( \boldsymbol{a,b} \right)\boldsymbol{,0)\cdot dlog(a)\cdot dlog(b)}$ |
| **Same as SUM_SYN_ANT_WEIGHTED, but synergistic effects are not considered.** | |
| SYN_SPREAD | $\sqrt{\boldsymbol{SYN\_SUM}\boldsymbol{/SYN\_MAX}}$ |
| **A measure of synergy spread in logarithmic space. To differentiate for instance between a spread over a square of 2 log x 2 log versus 1/2 log x 1/2 log.** | |
| ANT_SPREAD | $\sqrt{\boldsymbol{ANT\_SUM}\boldsymbol{/ANT\_MAX}}$ |
| **A similar measure of spread as SYN_SPREAD, but for antagonism.** | |
| SYN_AVERAGE_C1 | $\boldsymbol{10}^{\iint_{\boldsymbol{min}\left( \boldsymbol{a}>0 \right)\boldsymbol{,min(b}>0)}^{\boldsymbol{max}\left( \boldsymbol{a} \right)\boldsymbol{,max(b)}} \boldsymbol{loga\cdot max(S}\left( \boldsymbol{a,b} \right)\boldsymbol{,0)\cdot dlog(a)\cdot dlog(b)/SYN\_SUM}}$ |
| **Concentration value of drug 1 where synergy appears to be localized.** | |
| SYN_AVERAGE_C2 | $\boldsymbol{10}^{\iint_{\boldsymbol{min}\left( \boldsymbol{a}>0 \right)\boldsymbol{,min(b}>0)}^{\boldsymbol{max}\left( \boldsymbol{a} \right)\boldsymbol{,max(b)}} \boldsymbol{logb\cdot max(S}\left( \boldsymbol{a,b} \right)\boldsymbol{,0)\cdot dlog(a)\cdot dlog(b)/SYN\_SUM}}$ |
| **Concentration value of drug 2 where synergy appears to be localized.** | |
| ANT_AVERAGE_C1 | $\boldsymbol{10}^{\iint_{\boldsymbol{min}\left( \boldsymbol{a}>0 \right)\boldsymbol{,min(b}>0)}^{\boldsymbol{max}\left( \boldsymbol{a} \right)\boldsymbol{,max(b)}} \boldsymbol{loga\cdot min(S}\left( \boldsymbol{a,b} \right)\boldsymbol{,0)\cdot dlog(a)\cdot dlog(b)/SYN\_SUM}}$ |
| **Concentration value of drug 1 where antagonism appears to be localized.** | |
| ANT_AVERAGE_C2 | $\boldsymbol{10}^{\iint_{\boldsymbol{min}\left( \boldsymbol{a}>0 \right)\boldsymbol{,min(b}>0)}^{\boldsymbol{max}\left( \boldsymbol{a} \right)\boldsymbol{,max(b)}} \boldsymbol{logb\cdot max(S}\left( \boldsymbol{a,b} \right)\boldsymbol{,0)\cdot dlog(a)\cdot dlog(b)/SYN\_SUM}}$ |
| **Concentration value of drug 2 where antagonism appears to be localized.** | |

*log()* refers to decimal logarithm

**Types of figures**

Several types of figures are provided. These are presented in the following table:

| **Single agent dose-response** | |
| --- | --- |
| Single agent dose-response data and fitted curve for drug A | 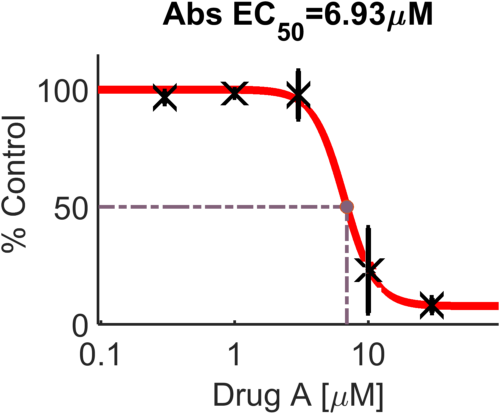 |
| Single agent dose-response data and fitted curve for drug B | 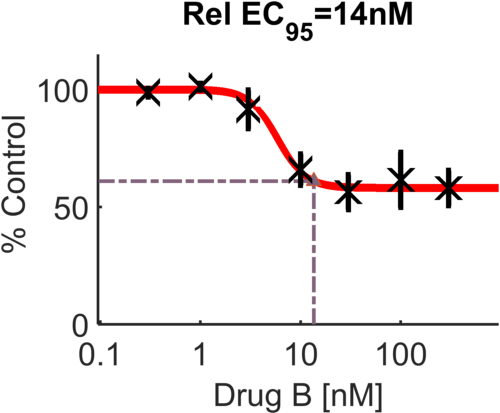 |
| **Combination dose-response** | |
| Combination dose-response data in matrix format | 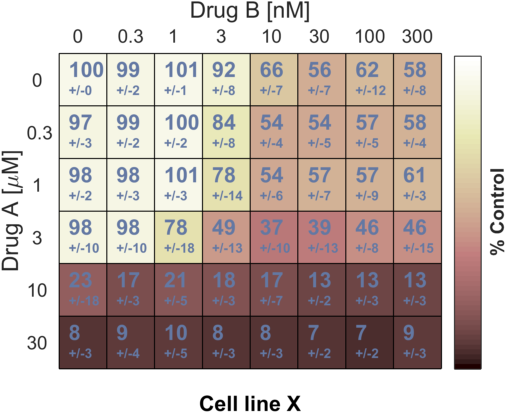 |
| Combination dose-response data visualized as a surface | 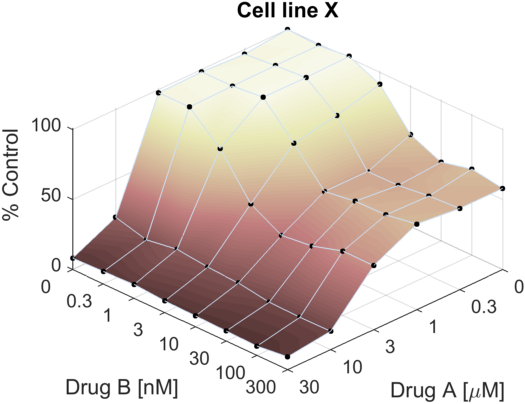 |
| Combination dose-response data visualized as contours, with 25%, 50%, 75% and 100% of control levels. | 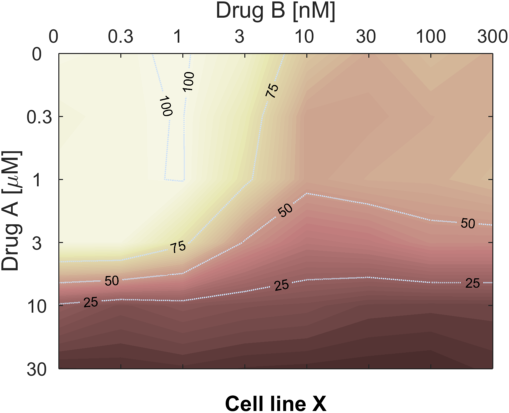 |
| B dose-response shift in presence of increasing concentrations of A | 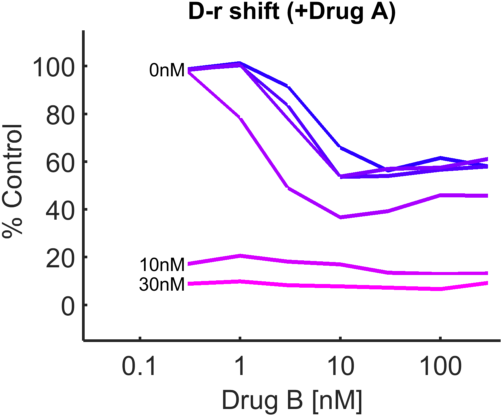 |
| A dose-response shift in presence of increasing concentrations of B | 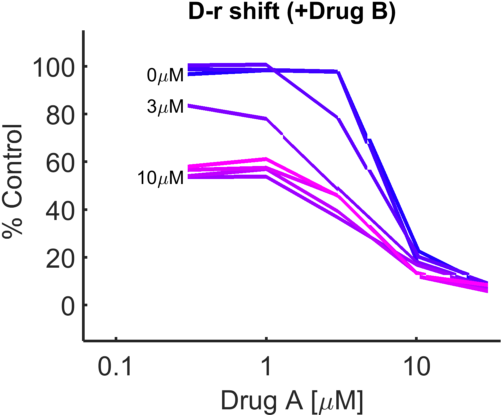 |
| **Reference modelled dose-response**  ***(Example using the Bliss model. Same type of graphic for other models)*** | |
| Modelled reference dose-response in matrix format. | 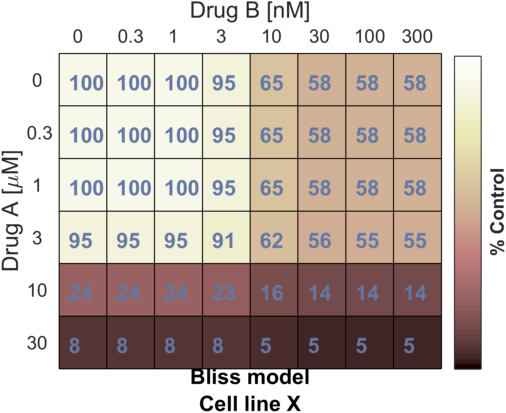 |
| Modelled reference dose-response visualized as a surface. | 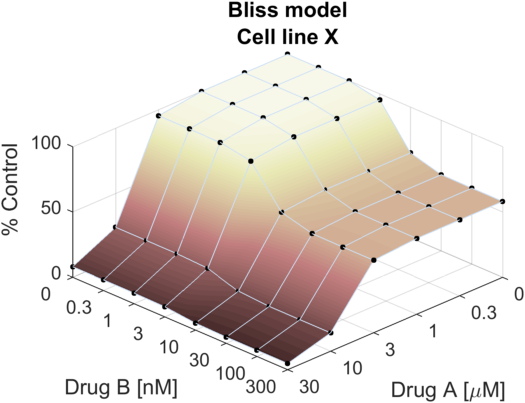 |
| Modelled reference dose-response visualized as contours, with 25%, 50%, 75% and 100% of control levels. | 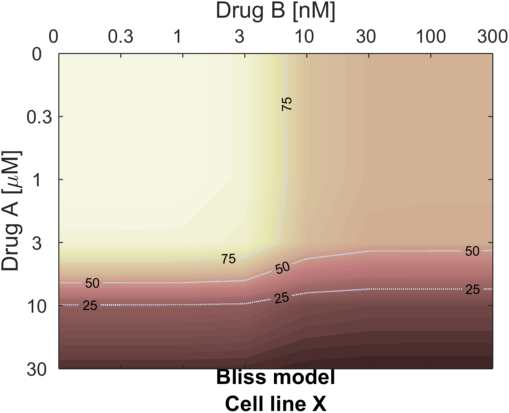 |
| **Synergy distribution**  ***(Example using the Bliss reference model. Same type of graphic for other models)*** | |
| Synergy levels in matrix format. Here the synergy distribution was derived from the combination dose-response and the reference dose-response. | 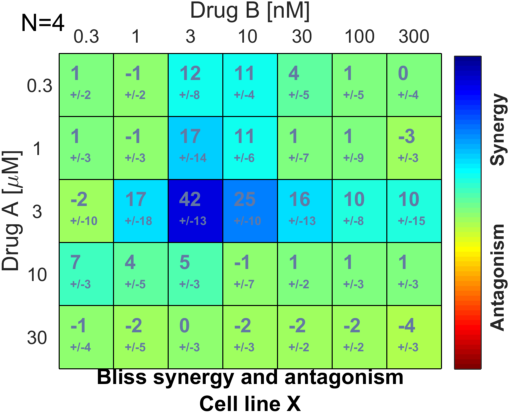 |
| Same graphical output as above but here synergy and/or antagonism levels are coloured according to the obtained score only if the result is significant following a one‑sample t‑test (* p<5x10^-2^_;_ **p<10^-3^, ***p<10^-4^; the number of replicates (N) is shown on the left top corner of the matrix display). This matrix is displayed if the “test significance” option is ticked. | 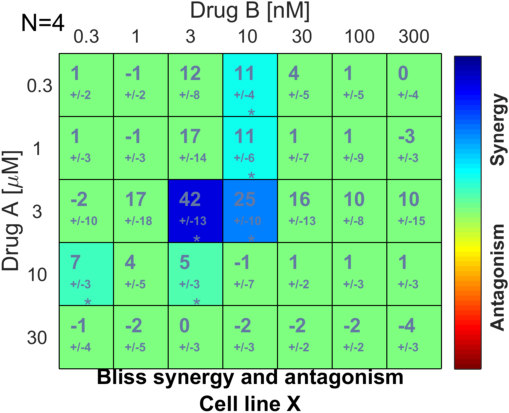 |
| Synergy levels visualized as a surface. | 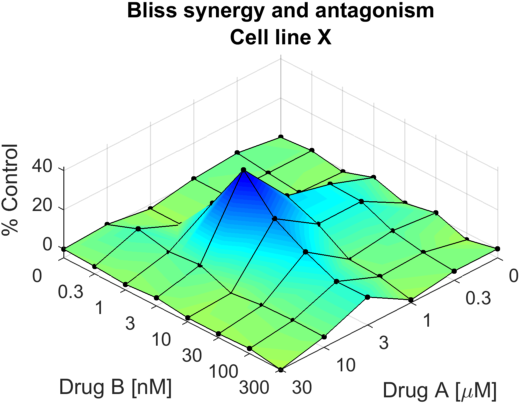 |
| Synergy levels visualized as contours, with isolines of synergy and/or antagonism shown for (when relevant): -100, -75, -50, -25, 0, +25, +50, +75 and +100%. | 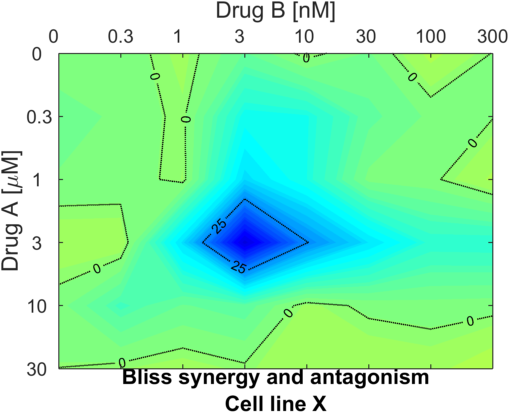 |
| **Combination dose-response and synergy** | |
| Mapping of the synergy levels on the experimental combination dose-response surface. | 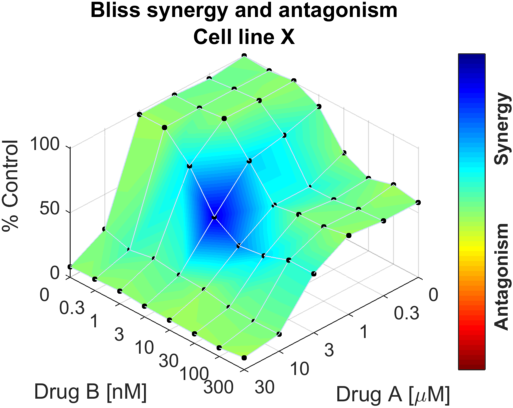 |

**Quality Assessment (QA) flags:**

The following flags are provided in the generated metrics.xls files

| **Flag** | **Meaning** |
| --- | --- |
| **0** | No data was found in a combination folder or NaN was found in a combination file. |
| **-1** | At least one of the measured drug effects was above 125% of starting cell count. This is unlikely to be genuine and a major experimental issue is suspected. |
| **-2** | No flag '-1' but measured effects below -10% were found. By definition, effects should always be positive because cell viability is being measured. Very small negative values are sometime encountered due to quantification problems at high concentrations. These were tolerated up to -10% below which major issues were suspected. |
| **-3** | No flags '-1' or '-2' but combination dose-response showed very strong fluctuations. The combination dose-response was smoothed and compared to the original non-smoothed version. If differences above 25% were found the experiment was flagged as measurements likely to be unreliable. |
| **1** | None of the previous problems were encountered. Data is supposed to be ok. |
